# Near-Infrared Irradiation Increases Length of Axial Pattern Flap Survival in Rats

**Published:** 2017-09-06

**Authors:** Yoshichika Yasunaga, Kiyoshi Matsuo, Yohei Tanaka, Shunsuke Yuzuriha

**Affiliations:** ^a^Department of Plastic and Reconstructive Surgery, Shinshu University School of Medicine, Matsumoto, Nagano, Japan; ^b^Clinica Tanaka Anti-Aging Center, Matsumoto, Nagano, Japan

**Keywords:** near-infrared, electromagnetic radiation, flap survival, surgical delay, rat

## Abstract

**Objective:** We previously reported that near-infrared irradiation nonthermally induces long-lasting vasodilation of the subdermal plexus by causing apoptosis of vascular smooth muscle cells. To clarify the possible application of near-infrared irradiation to prevent skin flap necrosis, we evaluated the length of axial pattern flap survival in rats by near-infrared irradiation. **Methods:** A bilaterally symmetric island skin flap was elevated under the panniculus carnosus on the rat dorsum. Half of the flap was subjected to near-infrared irradiation just before flap elevation with a device that simulates solar radiation, which has a specialized contact cooling apparatus to avoid thermal effects. The length of flap survival of the near-infrared irradiated side was measured 7 days after flap elevation and compared with the nonirradiated side. **Results:** The irradiated side showed elongation of flap survival compared with the nonirradiated side (73.3 ± 11.7 mm vs 67.3 ± 14.9 mm, respectively, *P* = .03). **Conclusions:** Near-infrared irradiation increases the survival length of axial pattern flaps in rats.

Distal flap necrosis is a problem that remains to be resolved in reconstructive surgery,^1^ and a great deal of effort has been expended to develop means of increasing flap survival length.^2^ Previously, we showed that near-infrared (NIR) irradiation nonthermally induced long-lasting vasodilation of the subdermal plexus by apoptosis of vascular smooth muscle cells in a rat model and that moderate sunburn after prolonged sun exposure prolongs inflammatory vasodilation.^3^ On the basis of these findings, we hypothesized that NIR irradiation may increase the survival length of skin flaps by vasodilation and thus prevent distal flap necrosis. To clarify the possible application of NIR irradiation to prevent skin flap necrosis, we evaluated whether NIR irradiation increases survival length of the random pattern part of extended axial pattern flaps in rats.

## METHODS

### Animals

Twelve male Wistar rats (*Rattus norvegicus albinus*) weighing 320 to 350 g were divided into a control group (n = 6) and an NIR irradiation group (n = 6). Experiments were performed in a temperature-controlled environment (24°C ± 1.5°C) under a 12-hour light-dark cycle, with free access to water and standard rat chow. All animals were treated humanely and in compliance with the recommendations of the Committee for Animal Experiments of Shinshu University (approval no. 230049). Animals were anaesthetized by intraperitoneal injection of a mixture of medetomidine hydrochloride (0.15 mg/kg), midazolam (2.0 mg/kg), and butorphanol tartrate (2.5 mg/kg) and were humanely killed by intraperitoneal injection of pentobarbital (120 mg/kg) upon completion of the experiments.

### Flap design and NIR irradiation

**Figure 1:**
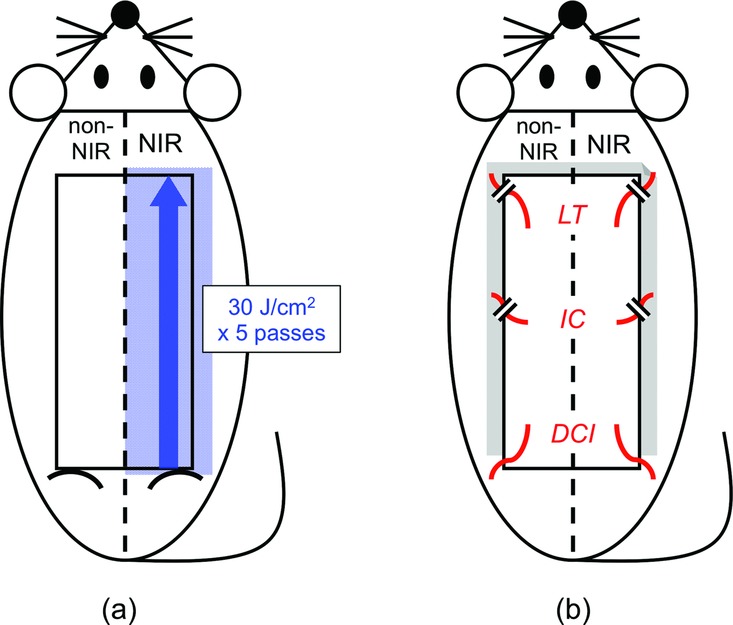
Rat skin flap model and NIR irradiation. (a) A 10 × 5-cm rectangular island skin flap was marked on the dorsum, which was symmetrical across the midline. For the NIR irradiation group, the right half was subjected to 5 passes of NIR irradiation at 30 J/cm^2^ just before elevation. (b) The flap was vascularized only by the bilateral deep circumflex iliac vessels. A polyethylene sheet was inserted between the flap and the donor site as an isolator and then the flap was sutured back in place. NIR indicates near-infrared irradiation; DCI, deep circumflex iliac vessels; IC, posterior intercostal vessels; and LT, lateral thoracic vessels.

**Figure 2:**
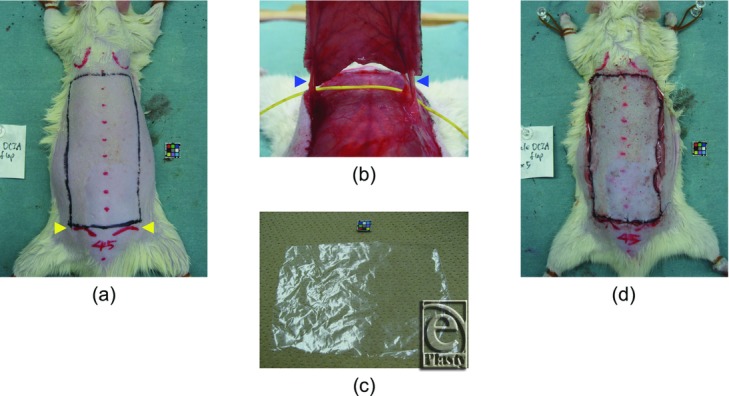
Flap procedures. (a) Flap design. Arrowheads (*yellow*) indicate the hip joints. (b) After flap elevation. Arrowheads (*blue*) indicate the deep circumflex iliac vessels. (c) Polyethylene sheet as an isolator (taken from a regular household bag). (d) After suturing back in place.

A 10 × 5-cm rectangular island skin flap, which was vascularized only by the bilateral deep circumflex iliac vessels, was marked on the rat dorsal skin. The flap bilaterally covered the 3 vascular territories of the lateral thoracic vessels, the posterior intercostal vessels, and the deep circumflex iliac vessels^4^ (Fig 1*b*) and was symmetrical across the midline. The caudal border of the flap was over the hip joints (Figs 1*a* and 2*a*).

The dorsal skin hair of the rats was removed using depilatory cream (Veet Rasera; Reckitt Benckiser Japan, Tokyo, Japan) before flap design to avoid selective photothermolysis to the hair follicles caused by NIR irradiation (Fig 2*a*).

For the NIR irradiation group, the right half of the flap (2.5 cm wide) was subjected to 5 passes of irradiation at 30 J/cm^2^ from the caudal border to the cranial border of the flap along the midline just before flap elevation (Fig 1*a*). NIR irradiation was generated using a broadband NIR source (Titan; Cutera, Brisbane, Calif). The device emitted an NIR spectrum ranging from 1100 to 1800 nm and filtering wavelengths between 1400 and 1500 nm, thus simulating solar radiation. This procedure allowed us to deliver NIR without the wavelengths that are strongly absorbed by water and hemoglobin and allowed the safe delivery of NIR energy deep into the tissue. The horizontal spot size of the irradiation was 10 × 30 mm (3 cm^2^), so the total energy delivered to each rat was 450 J (30 × 3 × 5) and the energy delivered to the right half of the flap was 375 J (450 × 2.5/3.0).

To avoid thermal effects, a sapphire contact cooling tip was set to a fixed temperature of 20°C to provide contact cooling. The sapphire block was cooled with fluids using a thermoelectric cooler, with the cooling fluids circulated by a pump and cooling system. This temperature-controlled sapphire window was used for pre- and parallel-irradiational cooling of the superficial layers, which further prevented excessive superficial heating.

In the control group, neither half of the flap was irradiated with NIR to assess individual variation of the experimental model.

### Dorsal axial pattern island skin flap vascularized by the bilateral deep circumflex iliac vessels

Just after NIR irradiation, the flap was elevated under the panniculus carnosus as an extended axial pattern island skin flap based on vascularity of the bilateral deep circumflex iliac vessels (Fig 2*b*). The flap was then sutured back to the donor site with interrupted 5/0 nylon sutures, with a polyethylene sheet inserted between the flap and the donor site as an isolator to prevent revascularization from the donor bed (Figs 1*b*, 2*c*, and 2*d*).

### Measurement of flap survival length on day 7

**Figure 3:**
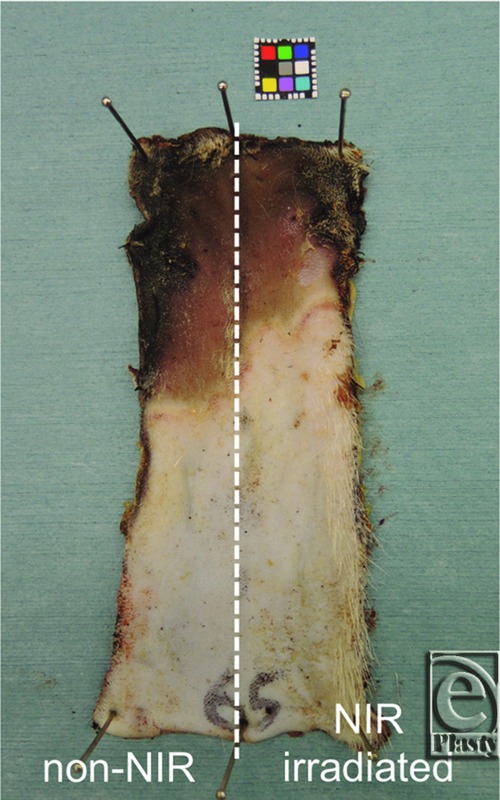
Flap survival on day 7. NIR irradiation group. NIR indicates near-infrared irradiation.

Seven days after NIR irradiation and flap elevation, the rats in both groups were killed to evaluate the flap survival lengths. The flap was cut off from the rat dorsum and pinned to a corkboard, and the length of surviving flap tissue on each side was measured (Fig 3).

### Statistical analyses

All values are expressed as means ± standard deviation. In each group, the differences in survival length of both sides of the flap were examined for statistical significance using the paired *t* test. The difference between flap tissue survival lengths of both sides in the control group together and the nonirradiated side of the NIR irradiation group was examined for statistical significance using Student's *t* test. All data were analyzed using IBM SPSS Statistics version 23 (IBM, Armonk, NY). In all analyses, *P* < .05 was taken to indicate statistical significance.

## RESULTS

**Figure 4:**
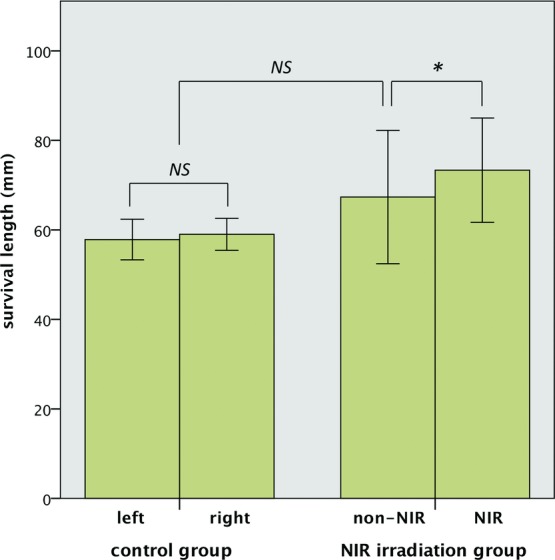
Results. In the control group, there was no significant difference between the left and right sides (57.8 ± 4.5 mm vs 59.0 ± 3.6 mm, respectively). In the NIR irradiation group, the length of flap survival of the NIR-irradiated side was greater than that of the nonirradiated side (73.3 ± 11.7 mm vs 67.3 ± 14.9 mm, respectively). There was no significant difference between the control group and the nonirradiated side of the NIR irradiation group (58.4 ± 3.9 mm vs 67.3 ± 14.9 mm, respectively). Error bar: mean ± 1 SD. NIR indicates near-infrared irradiation; *, statistically significant (*P* < .05); and NS, not significant.

The distal part of each flap became brown to black in color and was dehydrated with eschar formation due to impaired blood flow on day 7. In the control group, there was no significant difference in length of flap survival between the left and right sides (57.8 ± 4.5 mm vs 59.0 ± 3.6 mm, respectively). The length of surviving flap tissue on the 2 sides taken together was 58.4 ± 3.9 mm, and the coefficient of variation (standard deviation/mean) was 6.7%. In the NIR irradiation group, the flap survival length of the NIR-irradiated side was greater than that of the nonirradiated side (73.3 ± 11.7 mm vs 67.3 ± 14.9 mm, respectively; *P* = .03). In each individual, the length of surviving flap tissue on the NIR-irradiated side exceeded that on the nonirradiated side by 1 to 13 mm. No shortening of the surviving flap tissue was observed on the NIR-irradiated side compared with the nonirradiated side. There was no significant difference in survival length between the control group and the nonirradiated side of the NIR irradiation group (58.4 ± 3.9 mm vs 67.3 ± 14.9 mm, respectively) (Fig 4).

## DISCUSSION

The results of this study indicated that NIR irradiation increased the survival length of the irradiated half of extended axial pattern flaps in rats. We did not simultaneously perform histological examinations because we reported previously that NIR irradiation at 40 J/cm^2^ using the same device to the center of the dorsal portion of rats, which was equivalent to approximately 8.75 hours of sunbathing in North America,^5^ nonthermally induced long-lasting vasodilation by causing apoptosis of vascular smooth muscle cells^3^ and affected subcutaneous adipocytes and bones.^6^ NIR irradiation at 30 J/cm^2^ in this study appeared to nonthermally induce long-lasting vasodilation of the subdermal plexus by causing apoptosis of smooth muscle cells, resulting in increased flap survival length. This is the first report regarding nonsurgical delay with electromagnetic radiation (EMR) that required neither a waiting period before flap elevation nor multiple irradiation procedures.

In this study, no erythematous change was observed on the NIR-irradiated skin. Although erythema is caused by increased blood flow in superficial capillaries in the dermis, we previously reported that vasodilation caused by NIR irradiation was observed in the subdermal plexus rather than in the dermis.^3^

The vasodilation by NIR irradiation is long-lasting in comparison with thermal vasodilation but in fact is not permanent. We reported that cross-sectional areas of the lumen of the subdermal plexus on the rat dorsum were abruptly dilated by NIR irradiation at postirradiation day 7 and showed a gradual shrinkage thereafter.^3^ Statistically significant increases in the cross-sectional areas were observed at days 7 and 30 but were not observed at days 60 and 90 compared with nonirradiated controls. Therefore, it is unnecessary to worry about adverse effects of permanently dilating the subcutaneous blood vessels.

**Figure 5:**
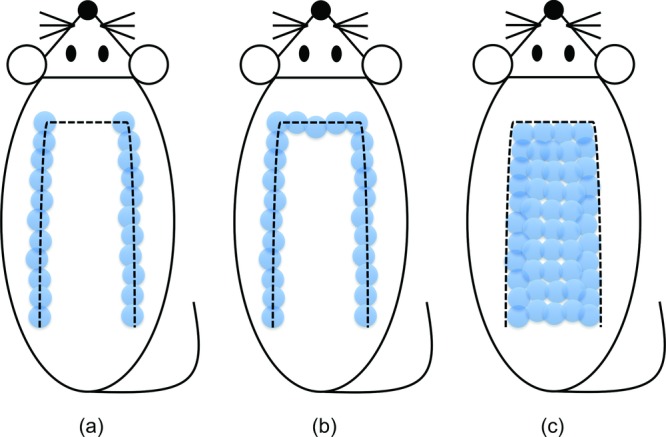
Two different concepts of EMR-assisted nonsurgical delay. (a), (b) Perimeter irradiation concept. EMR was applied along the longitudinal borders (a) or the longitudinal and distal borders (b). Dashed line indicates a planned flap. Each circle (*light blue*) indicates a single EMR shot. (c) Whole-surface irradiation concept. EMR was applied over the entire surface of a planned flap. EMR indicates electromagnetic radiation.

A great deal of effort has been directed toward developing methods to increase flap survival length with EMR, that is to say, EMR-assisted nonsurgical delay.^7^^-^19 Several types of EMR have been reported to increase flap survival length, such as gallium-aluminum-arsenide (Ga-Al-As) diode laser,^7^^,^^8^ neodymium:yttrium-aluminum-garnet (Nd:YAG) laser,^9^ tuneable dye laser,^10^ potassium-titanyl-phosphate (KTP) laser,^11^ far infrared irradiation,^12^ flashlamp-pumped pulsed dye laser,^13^^-^16 linear polarized infrared ray,^17^ erbium:yttrium-aluminum-garnet (Er:YAG) laser,^18^ CO_2_ laser,^18^ and intense pulsed light (IPL).^19^ Among these previous studies, we found 2 different concepts of EMR-assisted nonsurgical delay (Fig 5).

The first involves application of EMR only at the perimeter of a planned flap to disrupt blood flow along the longitudinal borders or the longitudinal and distal borders of the flap and to render the flap tissue ischemic, which is the same concept as in conventional surgical delay. Tuneable dye laser,^10^ KTP laser,^11^ flashlamp-pumped pulsed dye laser,^13^^-^15 Er:YAG laser,^18^ CO_2_ laser,^18^ and Nd:YAG laser^9^ have been used for this concept. Occlusion of the subdermal plexus at the lateral borders of the flap caused by EMR induced dilation and longitudinal rearrangement of the existing vessels, which resulted in delay.^15^ Although most types of EMR for perimeter irradiation were selective for oxyhemoglobin chromophores and were able to penetrate deep into the tissue, Reichner et al^18^ reported that the CO_2_ laser and Er:YAG laser, which penetrate only the most superficial layers, were as effective as surgical delay because tissue injury at the superficial layers generated chemical mediators that altered the flap microcirculation.

The other concept is to apply EMR over the whole surface of a planned flap, in which improvement of flap circulation is induced by angiogenesis. This is a new concept, and it has been implemented using Ga-Al-As diode laser,^7^ flashlamp-pumped pulsed dye laser,^14^^,^^15^ linear polarized infrared ray,^17^ and IPL.^16^ Although damage to the existing vascular architecture of the planned flap was thought to induce proliferation of blood vessels for a long time,^7^^,^^14^ the underlying mechanism was finally determined in 2003 by Erçöçen et al.^15^ They reported that whole-surface irradiation resulted in delay by inducing angiogenesis through activation and degranulation of mast cells.

In either concept, the common drawback is the need for a waiting period for rearrangement of the existing vessels or angiogenesis after EMR irradiation. Most of the lasers required about 14 days between irradiation and flap elevation,^9^^-^11^,^^13^^-^15 and Er:YAG laser and CO_2_ laser needed 7 days.^18^ IPL required 14 days in a mouse model,^19^ similar to most of the lasers in rat models.

In this study, we did not require a waiting period between NIR irradiation and flap elevation, so our NIR-assisted nonsurgical delay by whole-surface irradiation can be said as “instant” delay. We think that the ideal delay procedure is as follows: (1) no surgery but as effective as surgical delay; (2) single procedure; and (3) instant effect. NIR-assisted nonsurgical delay fills all 3 conditions, which makes NIR rays unique among EMR types.

Furthermore, the mechanism of delay phenomenon induced with NIR irradiation is totally different from any other types of EMR. In the perimeter irradiation concept, the delay phenomenon was induced by cessation of blood flow at the flap border as in surgical delay, whereas in the whole-surface irradiation concept, it was induced by activation and degranulation of mast cells. Unlike both of these concepts, NIR irradiation induces a delay phenomenon by apoptosis of vascular smooth muscle cells, as discussed earlier. Miyamoto et al^17^ reported that linear polarized infrared irradiation at wavelengths between 600 and 1600 nm, which has the highest capacity for tissue penetration among EMR types, improved the survival area of skin flaps, but they were unable to explain the mechanism. Although their infrared was polarized, its spectrum partially overlapped NIR used in our study (ranging from 1100 to 1800 nm except for wavelengths between 1400 and 1500 nm). Therefore, we propose that their findings resulted from vasodilation with the same mechanism as in our study.

With regard to timing of irradiation, Ga-Al-As diode laser^4^ and linear polarized infrared irradiation^14^ had advantages in increasing the flap survival length by irradiation even after flap elevation. However, they required daily irradiation for 5 or 7 days, and our NIR required only a single procedure for increasing flap survival length.

Our animal model was based on the Ohara et al^20^ rat dorsal paired island skin flap model, in which the survival areas appeared equal on both sides and the distal areas of necrosis were nearly symmetrical. We simplified their model to a single flap vascularized with bilateral deep circumflex iliac vessels because it is difficult to accurately divide the midline of the single flap into 2 symmetric flaps in terms of equal vascularity, which obviously affects flap survival length. An ideal animal experimental model in which to study flap survival is as follows: (1) small individual variation; (2) an internal control; and (3) use of an isolator to prevent skin-graft effects.

The McFarlane et al^21^ dorsal flap model was used most frequently to study flap survival, but their model was a random pattern flap resulting in unpredictable necrosis and had a single flap in each animal and therefore large numbers of animals were needed to obtain statistically significant results.^20^ The coefficient of variation of our model was 6.7%, which was much less than that of 20% in the McFarlane et al model^22^ and as low as that of 5.0% to 6.3% in the Ohara et al model. Thus, axial pattern models such as that of Ohara et al and ours have smaller individual variation than random pattern models and are more appropriate to study flap survival.

In our study, there was no significant difference in survival length of the 2 sides in the control group, indicating that both sides had equal areas of survival in this model. Moreover, the difference in survival length of the control group and the nonirradiated side of the NIR irradiation group was not significant, indicating that in our model, the nonirradiated side serves as an internal control to detect the effect of NIR irradiation on flap survival.

The distal random pattern part of a rat axial pattern flap is considered to be partially salvaged by the blood vessels emerging from its recipient bed, and flap survival of that part is thought to reflect that of a skin graft.^23^ The effect of recipient bed isolation with an artificial barrier on skin flap survival was evaluated in a rat model, and bed isolation was suggested for use in a study of rat skin flap survival.^24^ In our model, we used a polyethylene sheet as an isolator to prevent revascularization from the donor bed and therefore our model reflected the effect of NIR irradiation on flap survival accurately and sensitively. In conclusion, our model fulfilled all 3 conditions mentioned earlier and is one of the most practical models to evaluate flap survival in rats reflecting the effects of not only EMR types but also drugs, cytokines, or cells.

In terms of flap composition, our model is a myocutaneous flap, identical to the McFarlane et al^21^ flap. We elevated the flaps under the panniculus carnosus in rats because it was difficult to dissect the area without damaging the subdermal plexus. Because the point of action of NIR irradiation in this study was the subdermal plexus and not the panniculus carnosus as we reported previously,^3^ we consider the panniculus carnosus to be unnecessary as a carrier for blood supply and our NIR-assisted nonsurgical delay to be applicable to flaps of any composition if the subdermal plexus is preserved.

NIR-assisted nonsurgical delay is potentially applicable for reconstructive surgery in humans and has the potential to prevent distal flap necrosis. However, we cannot deny the possibility that NIR irradiation may increase the risk of skin cancer because it induces DNA strand breaks and cell death by apoptosis.^25^ While long-term exposure to NIR irradiation seems to be involved in photocarcinogenesis,^26^ NIR irradiation for nonsurgical delay requires only a single procedure and the total dose is quite low; 5 passes of NIR irradiation at 30 J/cm^2^ in this study was equivalent to no more than 32.8 hours of sunbathing in North America.^5^ Moreover, there has been no report of NIR-induced skin cancer to the best of our knowledge among the thousands of procedures performed worldwide with this device in aesthetic medicine. We would therefore consider the risk of NIR-induced skin cancer as limited if NIR-assisted nonsurgical delay were applied to humans.

In clinical practice, we routinely use this NIR-emitting device for skin tightening and usually start at 40 J/cm^2^ (based on our personal experience). The device was cleared for marketing to aesthetic professionals by the United States Food and Drug Administration on October 18, 2004 (501[k] no. K042165), as well as by Canadian, European, and Japanese authorities. Several clinical trials have already confirmed the efficiency and safety of this device in humans for skin tightening and relaxation of the corrugator supercilii muscle.^27^^-^33 The highest fluences used in each trial were ranged from 36 to 46 J/cm^2^, which were higher than the 30 J/cm^2^ employed in our study, and the most common and severe complication was blistering observed in 0% to 12% of patients due to incorrect technique. Therefore, we would advise starting at 30 J/cm^2^ if this device were used in humans for NIR-assisted nonsurgical delay and consider it possible to raise fluence to up to 46 J/cm^2^ if a delay phenomenon was not observed at a lower fluence.

We are currently planning to irradiate flaps in humans who require surgical delay but are unable to have repeated surgery for reasons such as age, complications, or duration of hospitalization. We are also considering flaps that are designed on skin with poor blood flow, including sites previously irradiated by radiotherapy, diabetic patient skin, and skin of the lower extremities, as possible candidates for NIR-assisted nonsurgical delay.

This study suggested that NIR irradiation may be useful to salvage flaps with impaired blood flow by irradiation after flap transfer and for the treatment of vasospasm in microvascular surgery. However, further studies are required to confirm these hypotheses.
